# Access to Drinking Water and Sanitation in Rural Kazakhstan

**DOI:** 10.3390/ijerph13111115

**Published:** 2016-11-09

**Authors:** Kamshat Tussupova, Peder Hjorth, Ronny Berndtsson

**Affiliations:** 1 Department of Water Resources Engineering, Lund University, Box 118, Lund SE-22100, Sweden; peder.hjorth@tvrl.lth.se (P.H.); ronny.berndtsson@tvrl.lth.se (R.B.); 2 Center for Middle Eastern Studies, Lund University, Box 201, Lund SE-22100, Sweden; 3 Department of International Cooperation and Bologna Process, Karaganda State Medical University, Gogol str 40, Karagandy 100048, Kazakhstan

**Keywords:** access to drinking water, sanitation, water services, rural Kazakhstan, SDG

## Abstract

The Sustainable Development Goals (SDGs) require nations to ensure adequate water supply for all. For Kazakhstan, this means that rural areas will need much stronger attention as they have been rather neglected in efforts to comply with the Millennium Development Goals (MDGs). This study aims to establish a baseline data concerning the current situation in villages that will need interventions according to the SDGs. The study was performed by means of questionnaires. The results should be seen as initial guidelines that can help to illuminate some of the uncounted challenges in future efforts to meet the SDG targets. As hardly any information exists about sanitation in rural Kazakhstan, the study essentially focuses on water services. The results show that 65% of rural dwellers want to connect and pay for the piped water supply. At the same time, about 80% have toilets outside their home. Consequently, the water program aiming at providing 80% of rural people with access to tap water from a centralized piped system will not be possible. However, by carefully managing the existing water supply and sanitation system in joint collaboration with the local users, significant progress can be made. The present results show the important first steps that need to be taken in this direction.

## 1. Introduction

Access to safe drinking water and sanitation is essential for both individual and population health as well as for quality of life and dignity. Indeed, improvement in water supply, sanitation, and hygiene has shown substantial influence on reduced water borne diseases such as diarrhea [1]. However, 663 million people worldwide still lacked improved drinking water sources [2,3], although the UN Millennium Development Goals (MDGs) for drinking water were achieved in 2010 on a global scale. However, several developing regions including Caucasus and Central Asia did not reach the MDG target. Moreover, with 2.4 billion still lacking improved sanitation facilities and 946 million practicing open defecation, the sanitation target was missed by almost 700 million people. Especially, there is a strong disparity between urban and rural populations. Eight out of ten people still without improved drinking water sources live in rural areas. The MDG progress report showed that the Kazakhstani urban population is 90% covered by piped water on premises, while only 28% of the rural people have access to piped water [2,3]. About 20% of the rural population in Kazakhstan actually has the same level of piped water coverage as sub-Saharan Africa.

The UN MDGs have now developed into the UN Sustainable Development Goals (SDGs) [4]. The SDGs present a continuation of the MDGs and a road map for how to ensure sustainable social and economic progress worldwide. Thus, the SDGs seek not only to eradicate extreme poverty, but also to integrate the three dimensions of sustainable development. An important difference between the MDGs and the SDGs is the change from a top-down to a bottom-up approach [5]. Thus, the SDGs emphasize gender goals, people’s participation, as well as local governance to reach sustainable development. The well-established links between poor sanitation and poor health mean that water supply must be viewed in connection with sanitation, and hygiene promotion as a coherent whole (WASH) [6,7].

Kazakhstan was the last of the Soviet Republics to declare independence after the dissolution of the Soviet Union in 1991 [8]. During the Soviet period, the poor living conditions experienced by much of the Kazakhstani population in the early part of the twentieth century were tackled by expanding access to essential services such as piped water. However, when the Soviet Union dissolved in 1991, the historically disadvantaged rural population still had limited access to water and the situation has become worse. Important elements of the state apparatus have been dismantled, leading to shortages of basic goods and services. Due to the transition from a socialistic to a market oriented system, the existing water supply system was not maintained and thus, has gradually deteriorated [9].

Information on access to drinking water and sanitation is based on official Kazakhstani statistics, data from the Joint Monitoring Program (JMP), and case studies provided by different researchers. According to [10], 17.3% of the rural Kazakhstani population had access to cold water on tap from the piped system and 2.8% had access to hot water on tap in 2001. The same survey showed that 92.2% of rural people had toilets outside the home, 7.5% inside the home, and 0.3% did not have access to toilets. According to the UNDP [11], the rural share of population corresponds to 43% and only 36% of them have access to a centralized water supply, 57.3% use groundwater (wells and boreholes), 2.6% use water from surface sources, and 4% drink delivered water. Previous studies have shown that only 2.8% of rural houses are connected to the sewage system. About 5% have in-house toilets, including 1.7% with toilets connected to local sewage systems, mostly wet pits [11]. This indicates that the sanitation level in rural Kazakhstan might be low.

Recent research has shown that there have been no significant changes in patterns of access to piped water during the period from 2001 to 2010, in neither rural nor urban areas in Kazakhstan [12]. In rural areas, access to piped water still remains about 29%. This situation is surprising because a massive governmental drinking water program for the rural areas was launched from 2002 to 2010 [13]. In any case, there is an urgent need to improve the water supply and sanitation conditions for rural areas in Kazakhstan. In addition, if rural water projects are to be both sustainable and replicable, an improved planning methodology is required that includes peoples’ desire to use different levels of services [13]. In particular, the people’s participation is crucially important. A new massive drinking water program in Kazakhstan has the aim to cover 80% of the rural people with access to tap water from a centralized piped system by 2020 [14]. Before executing such water supply projects, it is important to know the current situation of access to drinking water and sanitation services as well as whether or not people are willing to accept the new systems [15]. In line with this, the present study examines the current access to drinking water and sanitation services in rural areas of the Pavlodar region, Northern Kazakhstan. The aim is to estimate the willingness of different water users to connect to the piped water supply system to have tap water at home. The results are important since they can be used to predict the willingness to connect to public water supply and sanitation systems. Consequently, the results in this paper are important for the planning, policy development, as well as the management of new drinking water and sanitation programs in order to provide WASH for all.

## 2. Methodology

### 2.1. Area Description

**Figure 1:**
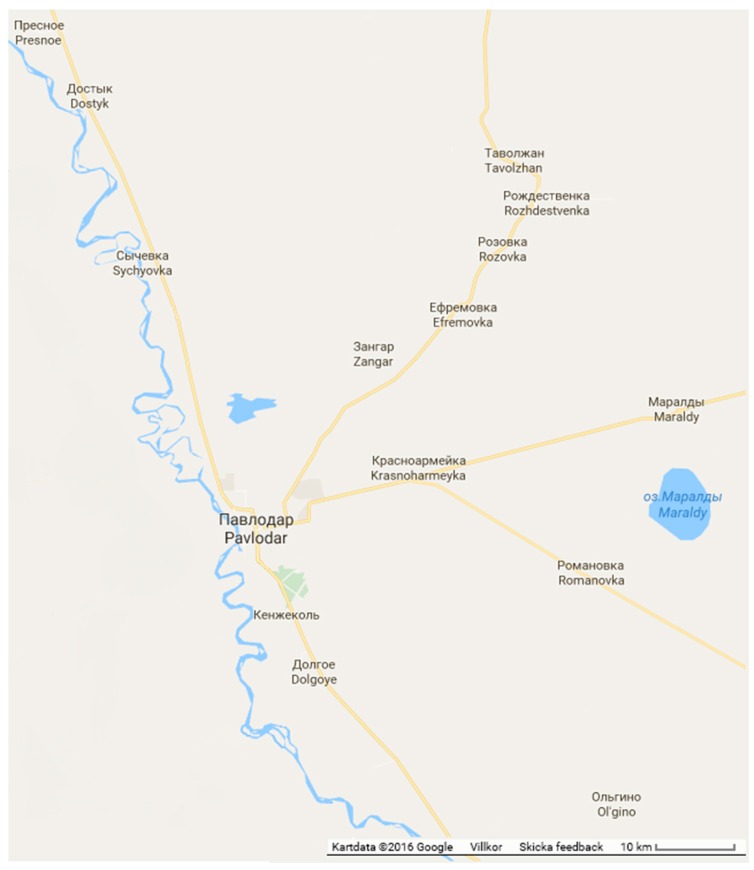
The experimental rural area around Pavlodar City in Kazakhstan.

The Pavlodar area is one of 14 regions in Kazakhstan. The region is located in the northeastern part of Kazakhstan within the Irtysh River Basin. It includes three cities and 412 rural districts (Figure 1). The population of the rural districts is about 270,000 persons. The area is dry with constant winds and about 250 mm of precipitation per year on average. The scant precipitation is unevenly distributed within the territory and between the seasons. Up to 80% of annual precipitation falls during the summer period. Most of the rainfall ends up as soil moisture and evapotranspiration. Average annual class A pan potential evaporation is about 800 mm. Thus, the available water resources of the region are mainly the Irtysh River and groundwater. Smaller rivers usually have a short spring discharge after snow melt before they dry up [16].

### 2.2. Water Supply Sources and Wastewater Management

The governmental decree on “Sanitarian-epidemiological requirements for water resources, drinking water sources, locations of cultural-domestic water use, and safety of water” regulates the water supply and drinking water sources in Kazakhstan [17]. Accordingly, main water supply systems are classified as centralized or decentralized (Table 1). The main difference between them is that the centralized water supply has a distribution system to provide water from the raw water source with or without treatment to the water user. The decentralized water supply system uses water directly from the raw water source with or without treatment. Consequently, centralized water supply means water provided through pipes to households (tap water) or public standpipes according to Table 1. Protected boreholes and wells are considered decentralized water supply sources. In order to be classified as having access to drinking water, one of the water supply sources should be accessible within a 500-m distance from the household. Both groundwater and surface water are sources for the centralized system. However, groundwater is the most common raw water source for rural centralized systems in the region. Official statistics also entail water users consuming water delivered by truck. Delivered water is regarded as an unsustainable water supply source and perceived as a temporary solution. Complex Block Module (CBM) is water for sale. It is typically constituted by treated groundwater that is sold by private vendors at a kiosk in gallons. This type of water is used for drinking water purposes in villages where a public water source is not available. Official statistics do not distinguish decentralized water sources being used privately or publicly (shared) while this survey includes both private and public decentralized water sources (Table 1).

The Soviet State tried to provide rural people with drinking water and build systems that needed low capital investment and small cost for process equipment but considerably high operational costs [18,19]. The majority of these water supply systems were constructed during the period 1950–1980. After dissolving the Soviet Union, the new government had little accountability and, in some cases, no financial capacity to maintain the water distribution systems. This led to a rapid deterioration. Although a national rural water program was put in place during 2002–2010, the poor management of the program was a major problem that resulted in virtually no progress. Even though the water supply systems are in a deteriorated state, not maintained, and officially recognized as not being used, rural people may still use these systems. The main problem of today is thus often to supply rural areas with safe water in a degraded pipe system.

As mentioned above, the Soviet State provided the water supply system. The wastewater collection and treatment system was, however, the responsibility of the villagers. Thus, the rural wastewater collection differs from village to village. Usually, however, rural houses have an outside pit latrine as a toilet. Greywater is often collected in a septic tank. Sometimes the water distribution system is complemented with sewage collection pipes where greywater goes untreated to local cesspools or wetlands. At present, however, there is no reliable information on how the wastewater is managed in the different villages.

### 2.3. Sample Collection

The survey was performed in the rural area around Pavlodar City outlined in Figure 1. The area covers 5578 km^2^ and 37 rural villages. The survey was performed in three steps. Initially, a pre-survey included visits to two villages and interviews with village mayors, village council responsible, hydrogeologist in the area, and village inhabitants. The experiences from the pre-survey were used to jointly design a pilot study together with the above collaborators. As noted by, e.g., Grosh and Glewwe [20], it is important to involve a team of experts, including members of the organization implementing the household survey. In the pilot study, 10 villages were randomly selected for a study on willingness to pay for water supply. The results of the pilot study were reported by Tussupova et al. [7]. The rest of the villages, in total 27 rural villages of different size, were investigated in the present survey conducted during July–August 2013. Thus, due to the participation of the 10 first villages in the pilot study, these were not included in the present survey.

Through the above-mentioned close collaboration with the local municipalities, a questionnaire was designed and distributed to all households in the 27 villages and consequently collected by the village mayors. Due to the local rules for performing interviews, this was a necessary manner to collect interviews, which meant that the practicalities were beyond influence by the investigators. As a result, response rates came to vary significantly among different villages. Depending on village, the households had from several days to a few weeks to answer the questionnaire. Interviews represent households and not individuals. Interviews were performed with the head of the households. The response rate was about 42%, ranging from 4% to 100% in each village (Table A1). Altogether, 2570 questionnaires covering 8493 persons in the area were collected. The objective of the survey was not to investigate conditions in individual villages but instead to get an overall picture of the access to water supply and sanitation in a larger representative area. Since the response rate varied significantly among different villages, the results should be seen as initial guidelines that can help to illuminate some of the uncounted challenges in future efforts to meet the SDG targets. The reasons for not receiving a higher response rate than 42% may have been: during the sampling period, some of the respondents were on summer work in the field and may not have been at home during the questionnaire distribution; some respondents might have had other reasons for not replying. In order to estimate the population-based representative sampling, Equation (1) was used to calculate the margin of error [21]:

(1) $$n\geq N/\left(1+e^{2}N\right)$$

where *n* is the sample size, *N* is the population size, and *e* is the margin of error denoting the allowed probability of committing an error in selecting a small representative sample size. According to Equation (1), the margin of error *e* for the survey is less than 0.02%, hence, the overall results can be viewed as statistically highly significant.

The questionnaire contained enquiries regarding the water source for drinking water, its perceived quality, reliability, time spent for collection, water treatment methods together with access to toilets, socioeconomic, and demographic characteristics. The data were analyzed using SPSS Version 22.0.

## 3. Results and Discussion

### 3.1. Description of the Households

Table 2 shows a general description of the investigated households. It is important to establish the general socioeconomic and demographic characteristics of the households since safe access to water and sanitation to a major extent is a question of socioeconomic conditions [22,23,24]. Most of the respondents were women (64%). This perhaps visualizes that women perceive water quality and sanitation as more important as compared to men. The span of age distribution was broad among the respondents. The migration in the area is quite low and most households have lived in the area for more than 5 years. The most common household structure is two adults and two children with an average of about 3.5 persons per household. A total of 96% of households do not include more than six persons.

The average monthly household income was 52,057 KZT with a standard deviation of 36,091 KZT (150 KZT around 1 USD as of January 2012). Probably, the notion of household income perception is a better description of household income. This displays how much the household can afford for a certain income. As shown by [10], there is a tendency in Kazakhstan to perceive the income better than before. This indicates that the economic situation of the households is improving.

### 3.2. Overview of Access to Drinking Water and Sanitation

**Figure 2:**
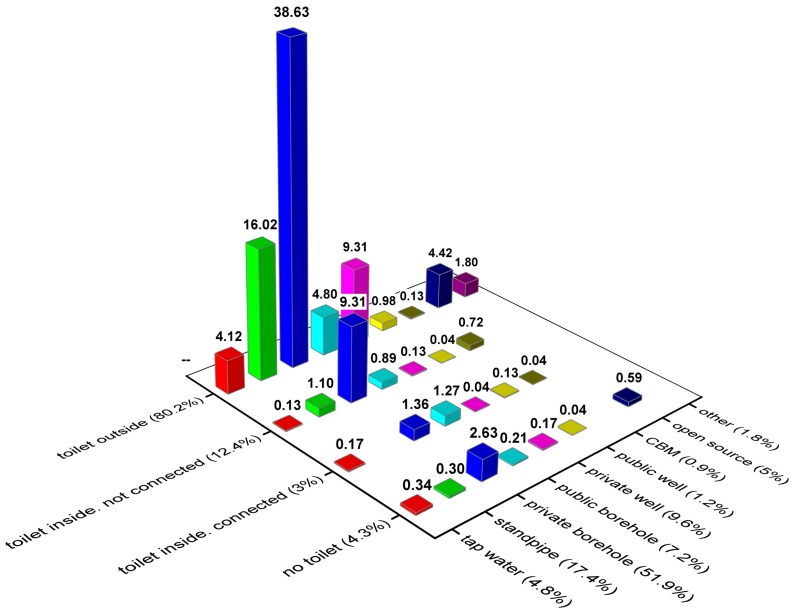
Rural users’ water supply source depending on toilet situation (%).

Figure 2 shows the rural users’ water supply source depending on the toilet situation. As seen from the figure, more than 80% of the respondents have toilets outside their homes. Most of these (39%) take water from private boreholes. About 16% of them use standpipe water. A further 9% take water from private wells and 5% of rural users take water from open sources such as water directly from the Irtysh River. These users mainly live close to the river. In total, 15% have a toilet inside their homes and about 4% do not use a private toilet meaning that they use a public pit latrine. Only 3% have a toilet inside that is connected to a sewage system and 12% have toilets inside but are not connected to the sewage network.

The most common raw water source is private borehole (>50%). The second most common water supply source is a standpipe (about 7%). Standpipes can usually be found at every street crossing in the rural villages.

Mainly four villages represent households that use open water sources (5%). All of these are relatively close to the Irtysh River. These villages, either in the past or currently, have had access to a piped water supply system. This leads to the conclusion that the water supply situation is problematic in these villages.

### 3.3. Household Access to Sanitation

Three questions were posed regarding access to sanitation, namely: (1) no private toilet; (2) private toilet outside home; and (3) toilet at home either connected to the sewer system or locally collected to a septic tank (Figure 2). The majority (80%) have their toilets outside in the yard in the form of a pit latrine and they are not connected to a sewage system. This is usually a hole in the ground, up to a few meters deep, which is covered by a concrete slab. Only about 15.3% have their toilet inside the house. Moreover, those who have toilets at home mostly use septic tanks for the sewerage. No toilet means no access to private toilet, and the household most probably uses a shared toilet outside with no charge.

It is important that a management system be developed to provide safe sanitation for the rural areas. The SDG target 6.2. suggests to support and strengthen the participation of local communities for improving water and sanitation management. Thus, a key aspect of this management system is to build on local participation and needs. The sanitation system of today to a major extent separates toilet waste and greywater. Building on local solutions that preserve the advantages, such as possibilities to re-use the greywater, would decrease needs for large-scale and expensive public treatment plants. Thus, careful planning and management are needed for the next step in the sanitation development. The new SDG goes beyond access to basic facility and addresses the safe management of fecal waste along the sanitation chain. The SDG indicator “percentage of population using safely managed sanitation services” means the proportion of the population using different types of basic sanitation facilities such as flush toilets and pit latrines, which are not shared and safely disposed in situ or transported and treated off-site. The emphasis is not whether a flush toilet is connected to a sewer system or septic tank or if pit latrines are used but instead on safe disposal of the excreta in order not to pollute the environment. Thus, a more economically feasible alternative for a sustainable sanitation system may be to concentrate on safe excreta disposal for those who use pit latrines rather than building a new large-scale wastewater system.

### 3.4. Household Access to Drinking Water

The most common water source, used by more than half of the investigated households, is groundwater through a private borehole (Figure 2). Boreholes are generally 8 to 50 m deep with a diameter of 10–30 cm. Water is usually pumped by electricity and in rare cases by hand. Boreholes are usually covered with a plastic top. Some of the households have connected the standpipe to their homes and have tap water from the borehole. All villages except one use water from boreholes.

The second most common water source is standpipe water (17%). Standpipe water is groundwater distributed through pipes and obtained from a standpipe at street crossings. In some cases, the standpipes may not be controlled and officially closed for usage. In many cases, however, people continue obtaining water from them. Only villages that historically have had or at present have access to pipelines may use standpipe water.

Private wells are used by about 10% of the households. Wells are usually about 10 m deep with a diameter of 0.5–1.5 m. In most cases, wells are covered with a wooden top. Public boreholes are used by 7% of the households. All households in a village can use standpipes connected to public boreholes. Often, piped water supply is constituted by groundwater from public boreholes. In some cases, people still use water from public standpipes created during the Soviet era.

The number of households that use open source water or have access to tap water at home coming from a central water supply system is almost the same, about 5% in each category. Groundwater is the main source of water for the central water supply. Open source water is taken from the Irtysh River either directly by the households or delivered from the source by payment. It is common for piped consumers to return to open sources when the system fails to deliver. This shows that there is an obvious problem in these villages to access safe water and a non-functioning public water supply. Households in few villages only, use water from a public well. Several households may share the well. The basic construction is similar to a private well. 

Very few households use delivered water. A special tanker delivers this water usually for a fee. According to the law in Kazakhstan, the government is responsible for providing people with potable water. The local municipality usually provides delivered water to the households that do not have access to potable water. This is not a sustainable solution; however, it must be used when there is no other way to provide potable water. In some cases, households themselves order delivered water and pay extra for this. 

The CBM is an abbreviation for Complex Block Module that treats groundwater in a so-called local treatment plant. The water is sold in gallons and people collect it using their own containers. The cost for this water ranges between 20 to 40 KZT per 20 L. Bottled water is water that households buy only for drinking purposes. A small number of households uses other sources of water for drinking purposes.

### 3.5. Perceived Characteristics of Water Source

**Figure 3:**
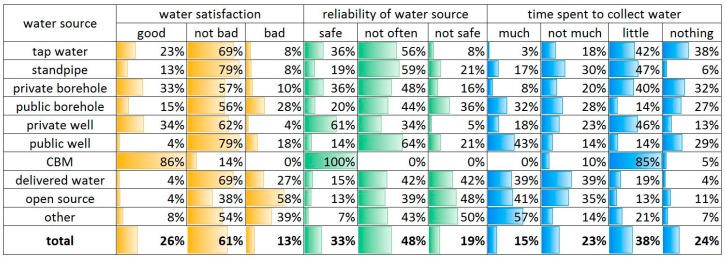
Perceived characteristics of the water source.

Three criteria were used to assess perceived characteristics of the water source, namely: (1) satisfaction with the water quality (such as turbidity, odor, and taste); (2) perceived safety of water; and (3) time spent to collect water (Figure 3).

The perceived water quality assessed the colour, smell, and taste of water. Most households (87%) perceived the quality of water as good or not bad and only a small portion was not satisfied with the quality (13%; Figure 3). Although the satisfaction with the quality of water appears relatively good, still for specific water users the satisfaction rate varies. The most unsatisfied are those who take water from the open and other sources (39%–58%), which is obvious. The most satisfied water users are those who buy water from CBM, although the portion of these water users is quite small. The next most satisfied are those who have a private water source such as private borehole and private well (33%–34%). Even standpipe users and public well users perceive the water quality as good or not bad (83%–92%).

Those who use tap water from the central water supply perceive the water as good (23%) or not bad (69%). Eight percent of the tap water users perceived the water quality as bad. The water from pipes often has a slight brownish colour and may appear to have some smell because of either old or not properly maintained pipes or contains a high mineral content from the groundwater.

In terms of reliability regarding the water source, the majority of households thought that the water source was not safe or not often safe (67%; Figure 3). This term is quite sensitive because it can be interpreted in several different ways. The question can both be interpreted as whether you are sure this water is safe to drink and whether you can obtain water from this source continuously regardless of season and other factors. In most cases, users with private water source find their water relatively safe (36%–61%). Except for the CBM water users, the majority of private well water users think their water source is reliable (61%). It is interesting to note that more than half of the standpipe and tap water users think that the water is not often safe, while one third of tap water users believe it is a safe and reliable source (36%). Only a small number of households think the tap water is not reliable (8%). This might be due to the fact that the water supply is given on a pre-determined time basis and that people may be ill-informed about this. Further studies are needed to elucidate these problems.

The question regarding time spent to collect water may not have felt relevant to all water users. However, this is an important aspect of water access. Those who use tap water may still have problems with temporal disruptions of the system. A majority of households did not spend any time or any time spent was very little. Those who use public source water generally spend considerable time to collect water (>32%). Those who use private borehole and centralized tap water spend less time than others. For private borehole water users, this might be due to connection problems to the house. Some borehole water users may not spend any time because water is connected to the home and used as tap water. A somewhat surprising result is that CBM water users spend little time. This may be due to the fact that the CBM plant is close to their house. This study did not adopt the distance to the source or exact time indicator. The pilot study showed that even some water users who have water at home could spend time on collecting water and the value of time can differ from person to person [8]. In addition, we find it more relevant to investigate to what extent people value their time to obtain the water.

Table 3 shows the results regarding whether households apply water treatment. This question was meant to decipher whether water users feel a potential risk to use water directly from the source without treatment. As seen from the table, overall, 45% of consumers generally do not treat the water in any way. About 37% boil the water before drinking. Only a small portion of respondents stated that they either let the water settle or use a filter. Again, those who use private water sources such as boreholes, well or tap water use water directly without treatment. A majority of tap water users (59%) do not treat the water before use. Also, those who use delivered water mostly use water directly without pre-treatment.

### 3.6. Importance of Water Supply to Household and Willingness to Connect to Piped Water System

Table 4 shows that a majority of households feel that water supply is important for the family. One may interpret this as the household feels either that there is an issue with the water supply or that the household generally thinks the water supply is important. In the questionnaire, we tried to make sure that the respondent understood the question as whether the household has an issue with the water supply and cares about its continuous improvement. In any case, in total, 72% think that the water supply is important.

**Figure 4:**
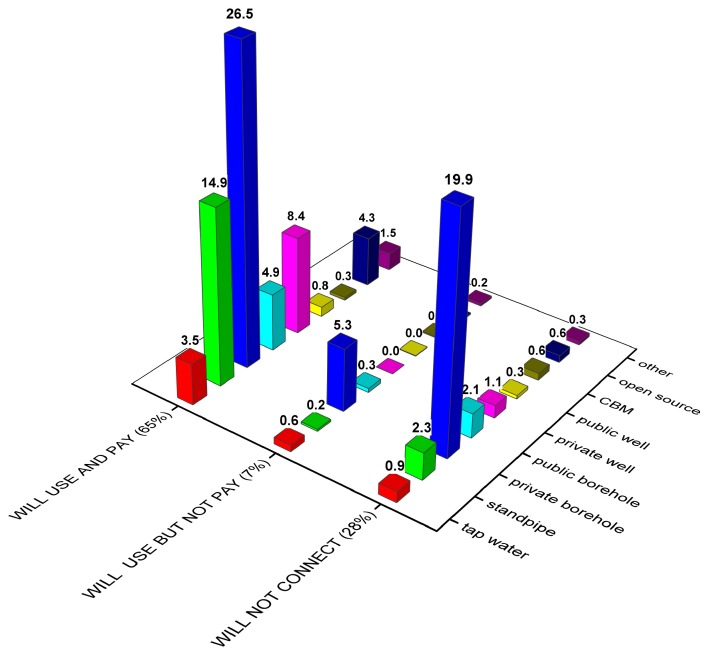
Willingness to connect to the piped water supply system depending on water user type.

Figure 4 shows the willingness to connect to the piped water supply system depending on water source type. As seen from the figure, a majority (65%) are willing to connect and pay for the water. About 28% of the households do not want to connect to piped water and about 7% would use it only if there is no fee (Figure 4). Among those who would not use piped water, the majority are private borehole users (20%). It should be noted though that this group is mixed. About 27% of private borehole users say that they are willing to connect to the piped water supply. One missing but interesting question to borehole water users is if water is connected from the borehole to the home. From the pilot study, it was seen that those who have connected water to the home and have a water boiler at home regardless of income have low or no willingness to connect to the piped tap water [7]. One of the reasons is that usually in rural areas cold water only is given through the pipes and households have to heat it by themselves using water boilers. Thus, if the households already have installed a convenient system using borehole water they are unwilling to use anything else.

Generally, in each category of water users a majority would like to connect to the piped tap water at home and pay monthly maintenance costs for the system. Regardless of perceived low quality of piped water, piped water users (tap and standpipe) still have a high willingness to connect. Particularly open source water users, as mentioned above, come from villages where they either used to have or still have access to piped water, and would like to connect and pay for the usage of tap water. This means that currently there are problems with the water supply system, but people still have a strong willingness to use piped water. One may assume that although there is a low satisfaction with the current tap water quality, still a well-functioning system in terms of water quality and absence of interruptions in the system seems attractive.

The households currently connected to the piped water system were asked whether they are willing to connect to the piped water system providing them with 24-h access to potable water. The answers confirmed that there is a high willingness to connect and pay for the continuous piped water supply.

It may be concluded that the majority of households are willing to connect to and pay for public water supply (65%). The major group that is satisfied with the present water supply situation is private borehole users (20%).

## 4. Conclusions

The current survey investigated access to drinking water and sanitation services as well as assessed households´ willingness to connect to the piped water system in 27 rural villages of the Pavlodar region. The results are important since they can be used to predict the willingness to connect to public water supply and sanitation and at what potential cost. Thus, they are important for the planning and fulfilment of the UN SDGs in Kazakhstan.

A majority of households (52%) use groundwater from private boreholes and, of these, 96% believe that the water is of good or not bad quality. About 17% of all households take water from the public standpipes and only 5% of them enjoy in-house tap water. About 5% use water from an unsafe water supply such as the Irtysh River. At the same time, 80% of people have private toilets (pit latrines) outside the house. About 15% have access to an indoor toilet and only 3% have access to and use a sewer system.

Despite efforts to provide people with potable water during the recently completed national water supply program, there is still a lack of access to tap water from the piped water supply system as well as access to safe sanitation. This may largely be explained by the severe lack of baseline data needed for targeting and designing improvements. Thus, there is a need for more ambitious data collection, as well as more selective and innovative ways to understand, share, and audit the data. Another reason is that interventions so far have been top-down. Furthermore, the responsible authorities need to appreciate that national drinking water programs need to be based on surveys of existing water and sanitation service, as well as a shift to more bottom-up and WASH oriented planning approaches. National drinking water programs need to include surveys of existing wastewater collection systems and need to collect and treat the wastewater centrally or on site. Thus, regardless of the type of basic sanitation, the safe management of fecal disposal is the core in a sustainable sanitation system.

A majority think that water supply is an important issue for the household and 65% would like to connect and pay for the piped water system. The fact that so many want to connect but still lack access to piped water indicates that there have been serious problems affecting the 2002–2010 drinking water supply campaign. The main water users who are reluctant to connect to a central water supply are those who have private boreholes (20%).

The results show that it will not be possible for Kazakhstan to reach 80% coverage of tap water from a centralized piped system to the rural people by 2020 according to the water program, whereas safe access to WASH for rural people is the most important. In any case, considerable progress can only be made by carefully managing the existing water supply and sanitation system in joint collaboration with the local users. Hence, we see the present results as an important first step in this direction.

## Figures and Tables

**Table 1:** Drinking water sources in northeast rural Kazakhstan.

Drinking Water Sources								
Centralized		Decentralized					Other	
tap water	standpipe	Borehole		Well		CBM	open source	other
		private	public	private	public			

CBM: Complex Block Module.

**Table 2:** Description of investigated households (SD = standard deviation).

Description	Percent	Mean
Respondent characteristics		
Sex of respondent: 1 = female, 0 = male		0.64
Age of respondent (min = 17, max = 90)		47 (SD 4.6)
Socio-economic characteristics of the household		
Living time for the household in the area:		
Less than 5 years	7%	
Between 5 and 10 years	14%	
More than 10 years	79%	
Number of people in household (min = 1 and max = 12). 90% of households contain up to 5 persons		3.45 (SD 1.6)
Family with children up to 18 years old: 1 = yes, 0 = no		0.51
Household monthly income in KZT * (min = 1000, max = 650,000, Median = 40,000)		52,057 (SD 36,091)
Household income perception		
Very good	2%	
Good	19%	
Satisfactory	70%	
Bad	8%	
Very bad	1%	

* 150 KZT around 1 USD as of January 2012.

**Table 3:** Household water treatment (percentage of households giving each response).

Water Source	Sample Size	No	Filter	Boiling	Settling	Other
Tap water	111	58.6	5.4	30.6	4.5	0.9
Standpipe	361	21.9	5.0	67.0	5.5	0.6
Private borehole	1155	51.8	14.2	27.4	6.5	0.2
Public borehole	153	37.9	6.5	47.1	8.5	0.0
Private well	219	50.7	5.9	33.3	9.6	0.5
Public well	26	34.6	0.0	65.4	0.0	0.0
Complex Block Module	22	13.6	77.3	9.1	0.0	0.0
Delivered water	25	52.0	8.0	32.0	8.0	0.0
Open source	89	42.7	5.6	48.3	3.4	0.0
Other	18	50.0	0.0	44.4	0.0	5.6
Total	2179	45.1	10.8	37.4	6.4	0.3

**Table 4:** Importance of the water supply issue to the household.

Importance of the Water Supply Issue to the Household	Percentage
Absolutely not important	10
Not important	10
Between important and not important	8
Important	38
Very important	34

## Appendix A

**Table A1:** Collected samples from each village.

Village	Official No. of Households	Observed No. of Households	Percent of Questioned Households	Official Access to Water
Chernojarka	181	134	74%	Decentralized
Novochernojarka	439	319	73%	Decentralized
Sychevka	150	39	26%	Decentralized
Chernoreck	402	160	40%	Decentralized/Centralized
Dostyk	138	77	56%	Centralized
Karakol	64	51	80%	Decentralized
Efremovka	333	190	57%	Decentralized
Naberezhnaja	459	174	38%	Centralized
Zhana kala	163	29	18%	Decentralized
Aitym	79	5	6%	Decentralized
Novojamyshevo	485	287	59%	Decentralized
Krasnoarmeika	648	121	19%	Decentralized/Centralized/CBM
Akkuduk	34	34	100%	Decentralized
Bogdanovka	108	97	90%	Decentralized
Lugansk	479	105	22%	Decentralized/Centralized
Maraldy	152	150	99%	Centralized
Olginka	297	183	62%	Decentralized
Presnoe	300	89	30%	Decentralized
Maksimovka	57	8	14%	Centralized
Rozhdestvenka	230	10	4%	Decentralized
Rozovka	440	47	11%	Decentralized/Centralized
Koktobe	16	4	25%	Decentralized
Shakat	194	135	70%	Centralized
Tolubai	55	9	16%	Decentralized
Zaozernoe	45	17	38%	Decentralized
Korjakovka	53	24	45%	Decentralized
Zangar	128	72	56%	Decentralized
Total	6129	2570	42%	

CBM: Complex Block Module.
